# Bis(azido-κ*N*)bis­(quinolin-8-amine-κ^2^*N*,*N*′)iron(II) monohydrate

**DOI:** 10.1107/S2414314625002354

**Published:** 2025-03-19

**Authors:** Fatima Setifi, Zouaoui Setifi, Hans Reuter, Mohammad Hadi Al-Douh, Abderezak Addala

**Affiliations:** a Laboratoire de Chimie Ingénierie Moléculaire et Nanostructures (LCIMN) Université Ferhat Abbas Sétif 1 Sétif 19000 Algeria; b Département de Technologie Faculté de Technologie Université 20 Août 1955-Skikda BP 26 Route d'El-Hadaiek Skikda 21000 Algeria; c Chemistry, Osnabrück University, Barabarstr. 7, 49069 Osnabrück, Germany; d Chemistry Department, Faculty of Science, Hadhramout University, Mukalla, Hadhramout, Yemen; Purdue University, USA

**Keywords:** crystal structure, azide, iron(II), hydrogen-bonding, 8-amino­quinoline

## Abstract

In the hydrated title complex, [Fe(N_3_)_2_(AQ)_2_]·H_2_O (AQ is 8-amino­quinoline, C_9_H_9_N_2_), the Fe^II^ ion is coordinated in a distorted octa­hedral manner by two neutral, chelating AQ ligands and two anionic, monodentate azide (N_3_^−^) ions in a *syn*,*cis*-configuration. From the two AQ ligands, the pyridyl N atoms are opposite to each other and the amino groups *trans* to the azide ligands. Distortion results from different Fe—N bond lengths [2.112 (2)–2.231 (2) Å] and 〈(N—Fe—N)_*cis*_ [75.25 (6)–99.91 (7)°] and 〈(N—Fe—N)_*trans*_ [159.98 (7)–170.62 (7)°] bond angles. The water mol­ecule acts as the acceptor of hydrogen bonds with the NH_2_ groups of both AQ-ligands in one and the same mol­ecule, and as donor to the γ-N and α-N atoms of the azido ligands of two adjacent iron complexes. In addition, both terminal N atoms of the azido ligands are involved in hydrogen bonds with NH_2_ groups in neighboring iron complexes, so that the hydrogen-bonding pattern leads to a rod-like arrangement of the mol­ecules in the *b*-axis direction.

## Abstract 2

In the title compound, [Fe(N_3_)_2_(C_9_H_8_N_2_)_2_]·H_2_O or [Fe(N_3_)_2_(AQ)_2_]·H_2_O, the Fe^II^ ion is distorted octa­hedrally coordinated by two azide ions in a *cis* position with a *syn* orientation, while the two amino groups of the AQ ligands are in a *cis* and the two pyridiyl N atoms are in a *trans* position.

## Graphical abstract

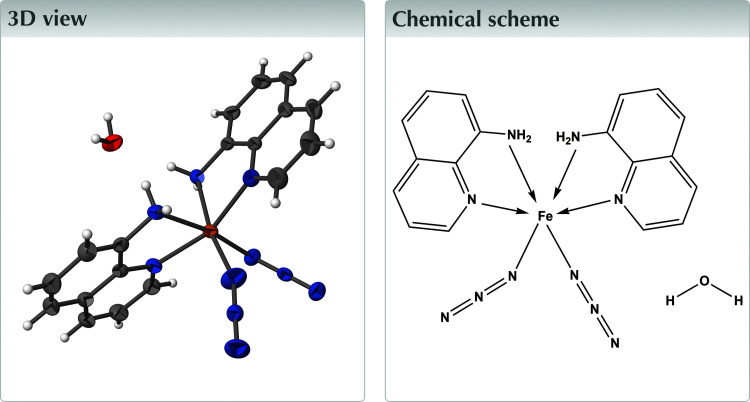

## Structure description

Pseudohalide compounds derived from transition-metal ions are of great inter­est from the perspective of their magnetic properties, rich mol­ecular architectures and for their topologies (Setifi, Ghazzali *et al.*, 2016 ▸, Setifi *et al.*, 2018 ▸, 2022 ▸; Merabet *et al.*, 2022 ▸). One of the pseudohalide ligands that has received much attention in the last decade is the azide [N_3_^−^] ion, partly due to its ability to produce a wide variety of coordination compounds with different nuclearities ranging from simple mononuclear to polynuclear species (Escuer & Aromi, 2006 ▸; Benamara *et al.*, 2021 ▸; Merabet *et al.*, 2023 ▸).

Up to now, mononuclear, octa­hedral iron(II) bis-azido complexes with bidentate Lewis bases LB_*NN*_ having the general composition Fe^II^(LB_*NN*_)_2_(N_3_)_2_, are only known for LB_*NN*_ = 1,10-phenanthroline (Miao *et al.* 2006 ▸), 4-amino-3,5-bis­(2-pyrid­yl)-1,2,4-triazole (Setifi *et al.* 2021 ▸), and quinolin-8-amine (Setifi, Moon *et al.*, 2016 ▸). Very recently, this class of compounds was expanded by hydrates with the monohydrate Fe(LB_*NN*_)_2_(N_3_)_2_·H_2_O where LB_*NN*_ = 2,2-di­pyridyl­amine (Setifi *et al.*, 2024 ▸).

Here we report on the monohydrate of the quinolin-8-amine complex, [Fe(N_3_)_2_(AQ)_2_]·H_2_O, revealing for the first time that hydrated as well as unhydrated forms of a specific azido iron(II) complex may exist and that the azido ligands in such complexes may have different orientations relative to each other. The compound was prepared under solvothermal conditions and its structure is described.

**Figure 1:**
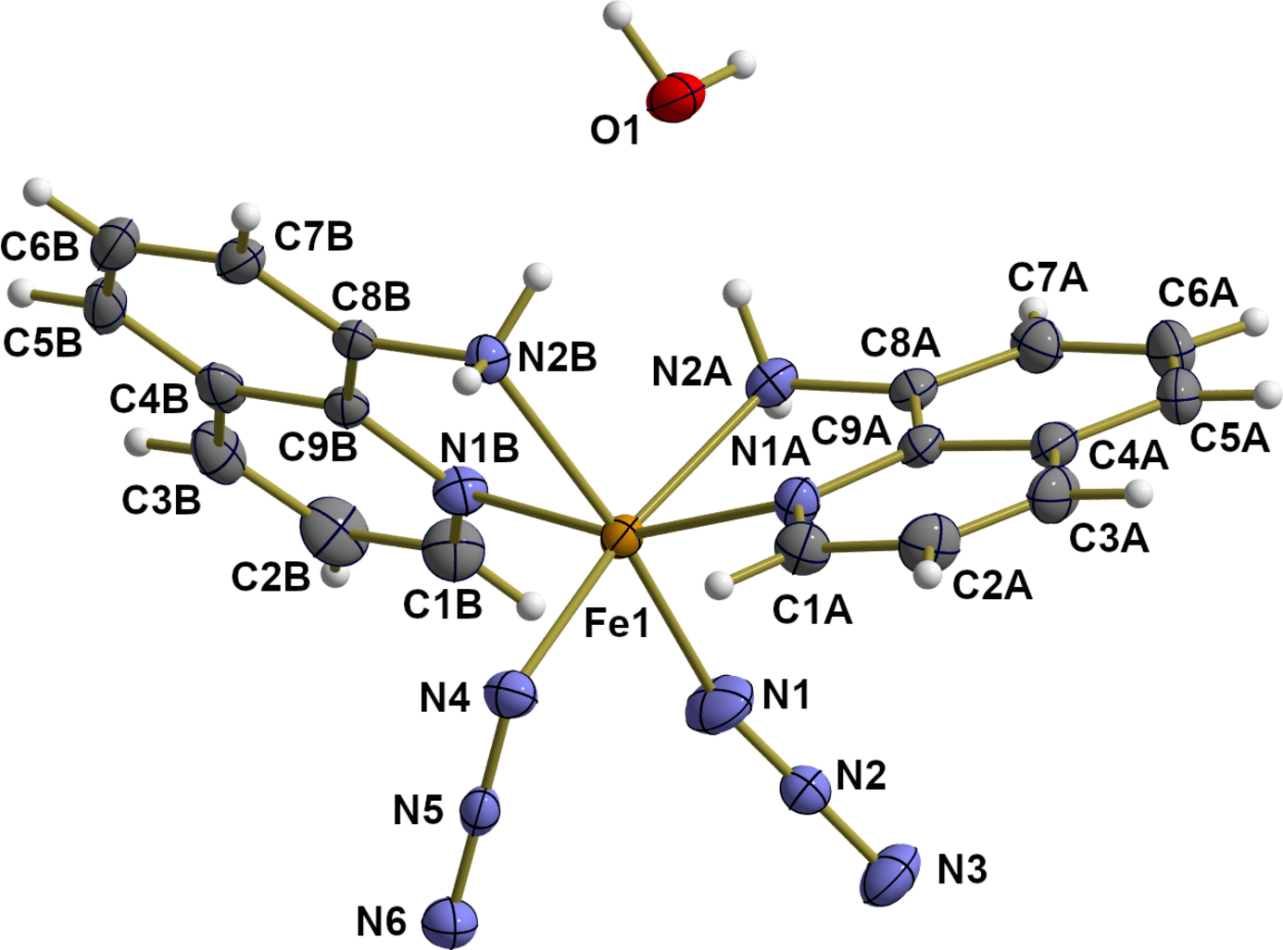
Displacement ellipsoid plot of the asymmetric unit of the title compound Fe^II^(LB_*NN*_)_2_(N_3_)_2_·H_2_O (LB_*NN*_ = AQ) showing the atom numbering. With the exception of the hydrogen atoms, which are shown as spheres of arbitrary radius, all atoms are drawn with displacement ellipsoids at the 40% probability level.

The title compound crystallizes in the ortho­rhom­bic space group *Pbcn* with eight formula units in the unit cell. The asymmetric unit therefore consists of one iron(II) complex and one water mol­ecule both with all atoms in general positions (Fig. 1 ▸). The overall composition of the complex corresponds to Fe^II^(N_3_)_2_(AQ)_2_ with two neutral, chelating Lewis base mol­ecules AQ = 8-amino­quinoline, and two monodentate azide ions, N_3_^−^, in a *cis* position. From the two AQ ligands, the pyridyl N atoms are *trans* and the two amino groups *cis* to each other.

**Figure 2:**
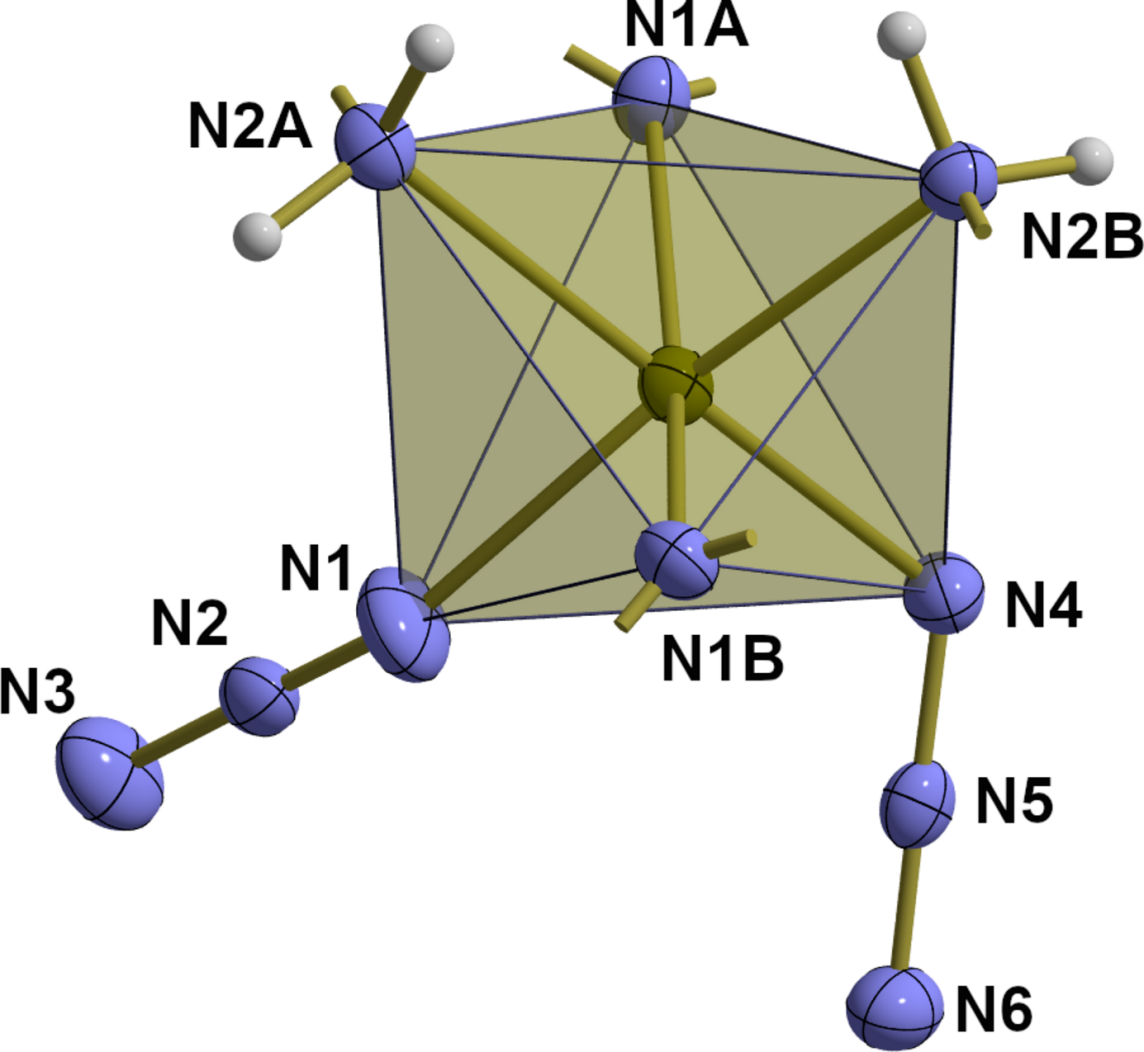
The {FeN_6_} octa­hedron in polyhedral representation, showing the *syn* orientation of both azido ligands. With the exception of the hydrogen atoms, which are shown as spheres of arbitrary radius, all atoms are drawn with displacement ellipsoids at the 40% probability level. The position of the carbon atoms attached to the nitro­gen atoms of the ligands are indicated as shortened sticks.

The Fe^II^ atom exhibits as usually a slightly distorted octa­hedral {FeN_6_} coordination (Table 1 ▸, Fig. 2 ▸). In contrast to the unhydrated compound (Setifi, Moon *et al.*, 2016 ▸), the two azido ligands have a *syn* orientation with an angle between them of 54.6 (6)°. A similar orientation was previously found in the tri­azane complex. Distortion results from different Fe—N bond lengths [*d*(Fe—N_azido_) = 2.112 (2)/2.142 (2) Å, < *d*(Fe—N_AQ_) = 2.177 (2)–2.231 (2) Å] and different bond angles [〈(N_Amine_—Fe—N_Quinoline_)_*cis*_ = 75.25 (6)/76.06 (7)°, 〈(N_Amine_—Fe—N_Azide_)_*cis*_ = 91.37 (7)/87.40 (8)°, 〈(N_Quinoline_—Fe—N_Azide_)_*cis*_ = 92.83 (8)–99.91 (7)°, 〈(N—Fe—N)_*trans*_ = 170.62 (7)/159.98 (7)/168.94 (8)°].

**Figure 3:**
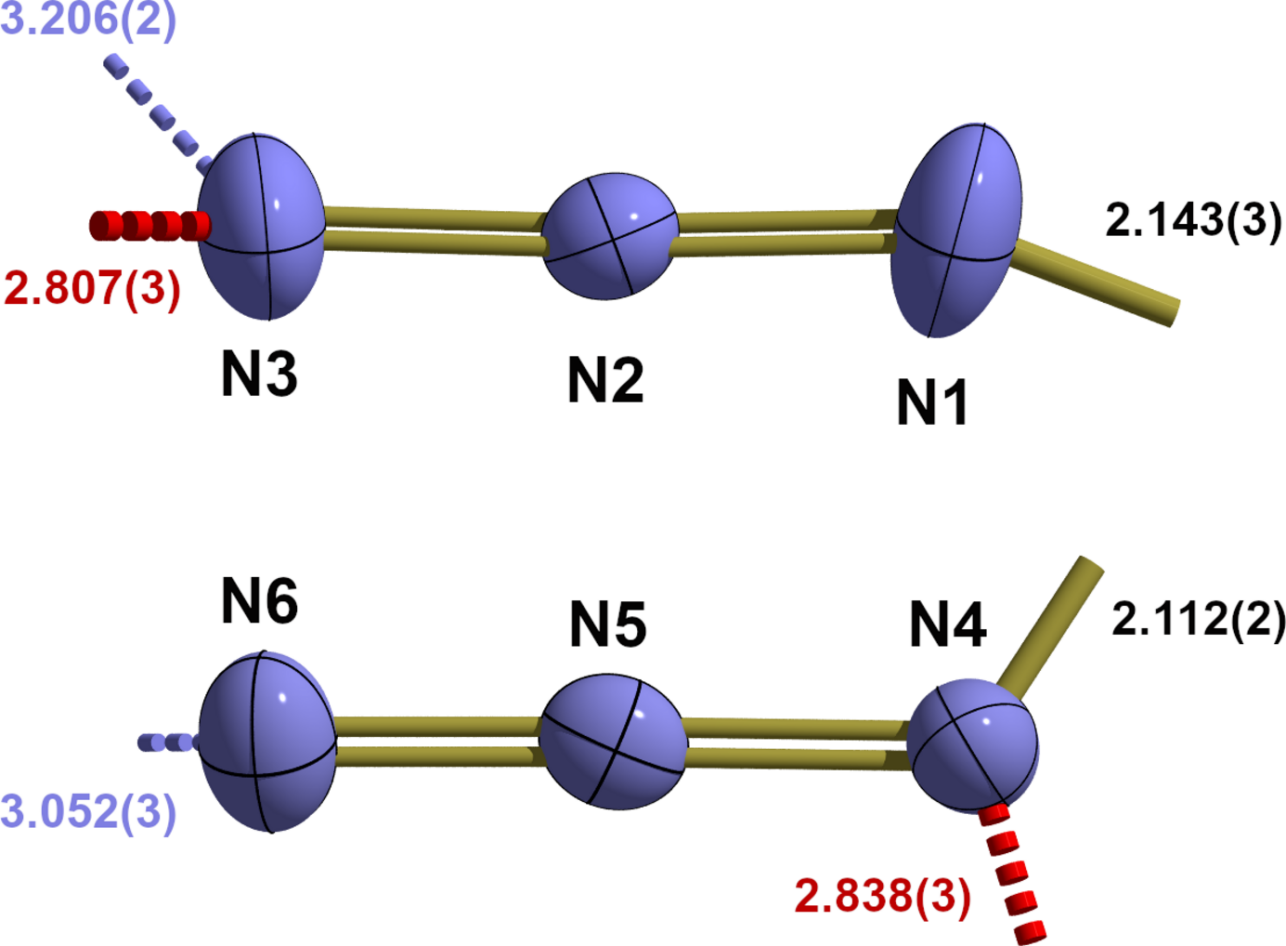
Displacement ellipsoid plot showing the two azido ligands in the iron(II) complex of the title compound in detail, with selected bond lengths (Å), hydrogen bonds [dashed, shortened sticks, *d*(*D*⋯*A*) in Å, O—H⋯N = red, N—H⋯N = blue] and dative bonds (shortened sticks) to the central iron atom. With the exception of the hydrogen atoms, which are shown as spheres of arbitrary radius, all atoms are drawn with displacement ellipsoids at the 40% probability level.

Both azido ligands are slightly bent [177.3 (3)/179.2 (2)°] with N—N bond lengths [1.159 (3)–1.202 (2) Å] typical for formal N=N double bonds with the longer one to the metal-coordinating N atom. They are different to some extend because of different coordination modes: in the first azido ligand (N1–N3) the metal-coordinated nitro­gen atom is additionally involved in a hydrogen bond (Table 2 ▸) to a hydrogen atom of the water mol­ecule and the terminal nitro­gen atom in a hydrogen bond to a NH_2_ group of AQ(B), while in the second azido ligand (N4–N6) the terminal nitro­gen atom N6 is involved in two hydrogen bonds, one to a hydrogen atom of a the water mol­ecule and second one to the hydrogen atom of a NH_2_ group of AQ(A) (Fig. 3 ▸).

**Figure 4:**
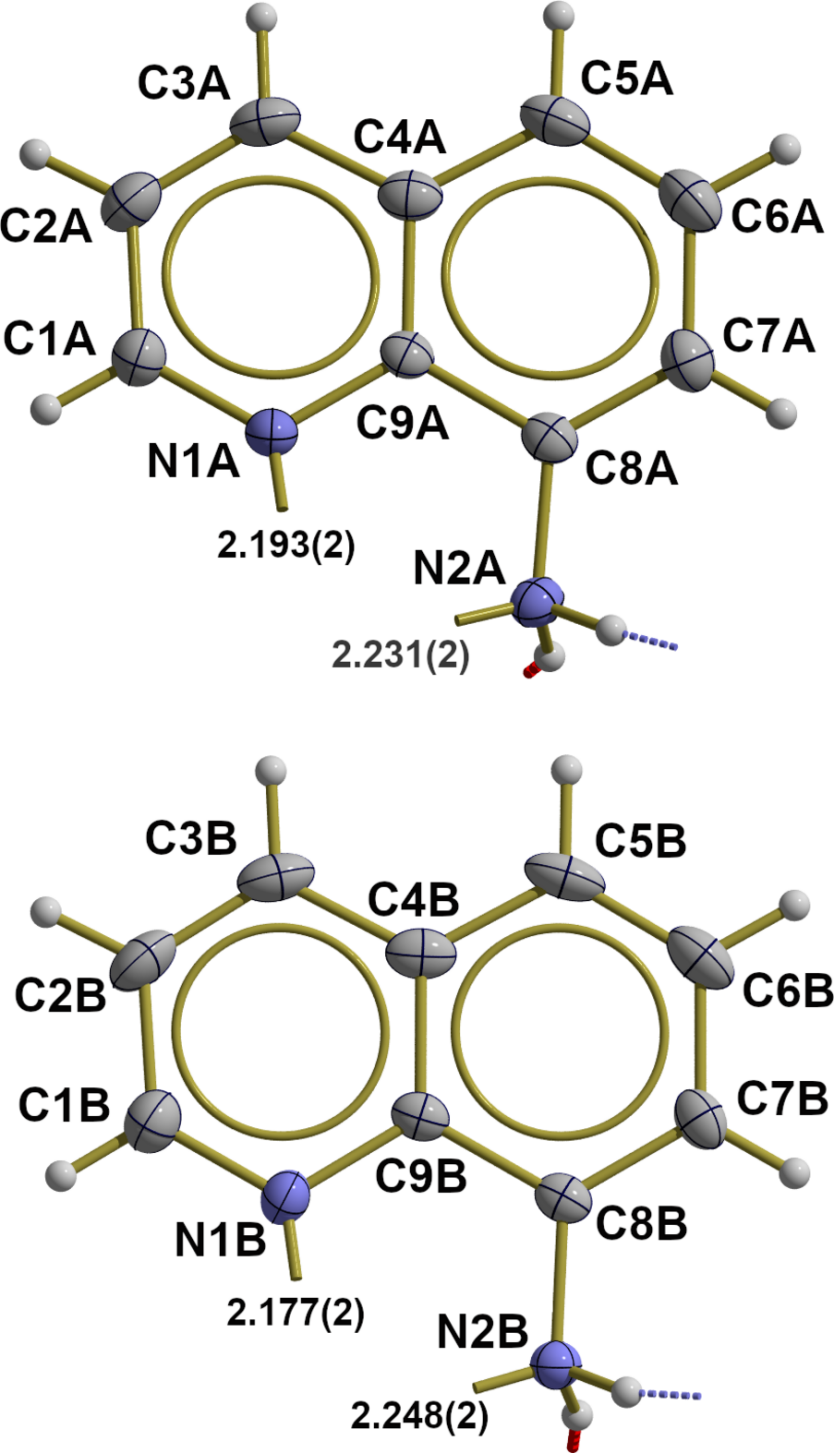
Displacement ellipsoid plot models showing the two 8-amino­quinoline ligand mol­ecules in the iron(II) complex of the title compound in detail, with selected bond lengths (Å), bond angles (°), and hydrogen bonds (O—H⋯N as red, N—H⋯N as blue dashed lines). With the exception of the hydrogen atoms, which are shown as spheres of arbitrary radius, all atoms are drawn with displacement ellipsoids at the 40% probability level.

**Figure 5:**
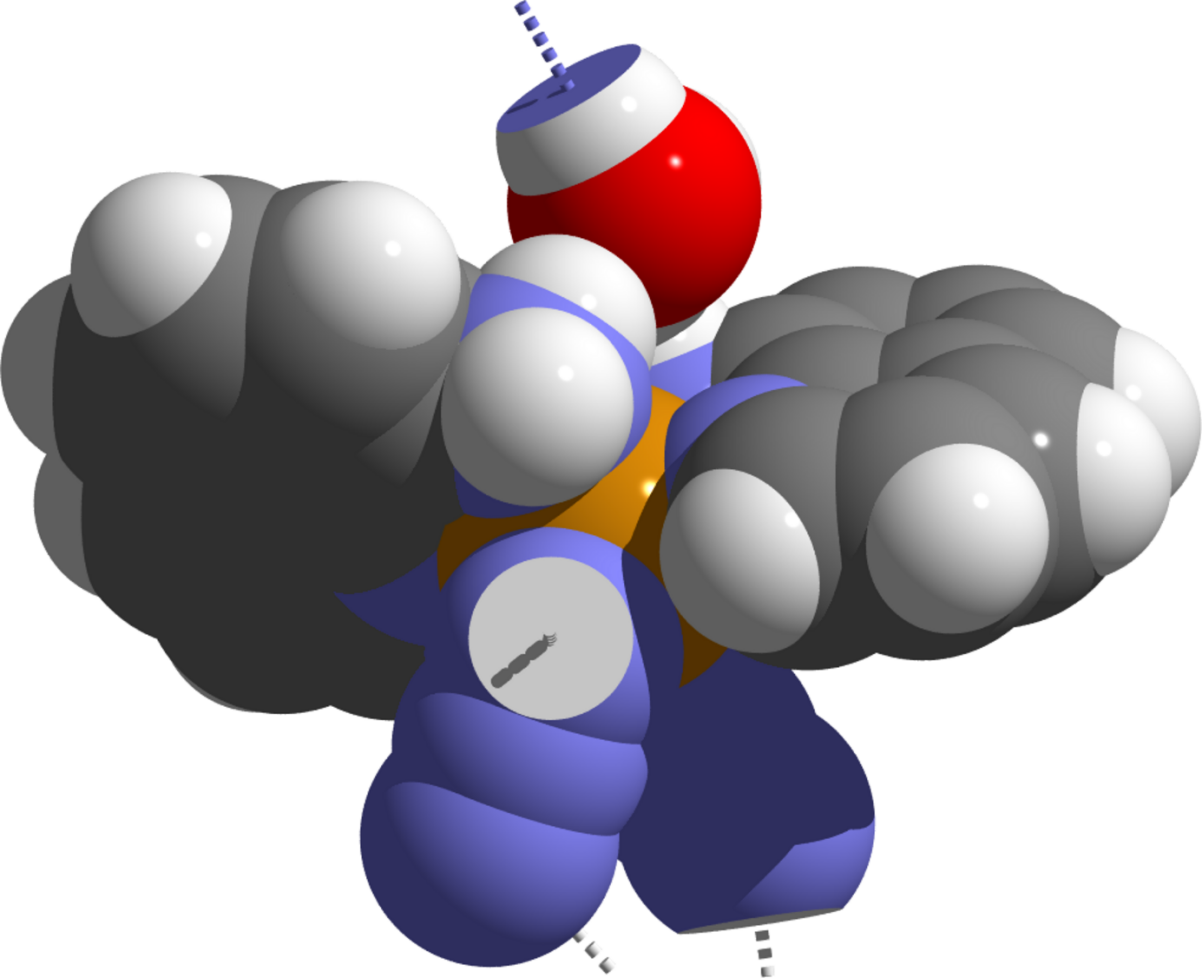
Space-filling model of one [Fe^II^(N_3_)_2_(AQ)_2_] complex mol­ecule and a water mol­ecule visualizing the hydrogen-bonding scheme (dashed lines). Atoms are drawn as single-colored or truncated, two-colored spheres according to their van der Waals radii and cut-offs based on the inter­section of the two spheres with cut-off faces showing the color of the inter­penetrating atom. Atom colors and van der Waals radii (Å) are as follows: H = white/1.10, C = gray/1.70, N = blue/1.55, O = red/1.52/ and Fe = orange/2.00.

N—C and C—C bond lengths and angles in the quinoline ring systems of the two ligands (labeled with suffixes *A* and *B*; Fig. 4 ▸) are comparable to those of the pure AQ mol­ecule (van Meervelt *et al.*, 1997 ▸) or the AQ mol­ecules in the unhydrated Fe^II^ complex (Setifi, Moon *et al.*, 2016 ▸) as are the bond lengths and angles of the attached NH_2_ groups. Both amine groups act as hydrogen donors in hydrogen bonds to the oxygen atom O1 of the water mol­ecule and to the terminal nitro­gen atoms of the azide ligands: N2*A* to the N6 atom of the second azide ion and N2*B* to N3 of the first one. The water mol­ecule also acts as a hydrogen donor in hydrogen bonds to the terminal nitro­gen atom N3 of the first azide ligand and to the iron coordinated nitro­gen atom N4 of the second one. Numerical details of the hydrogen bonds are summarized in Table 2 ▸ and visualized in Fig. 5 ▸.

**Figure 6:**
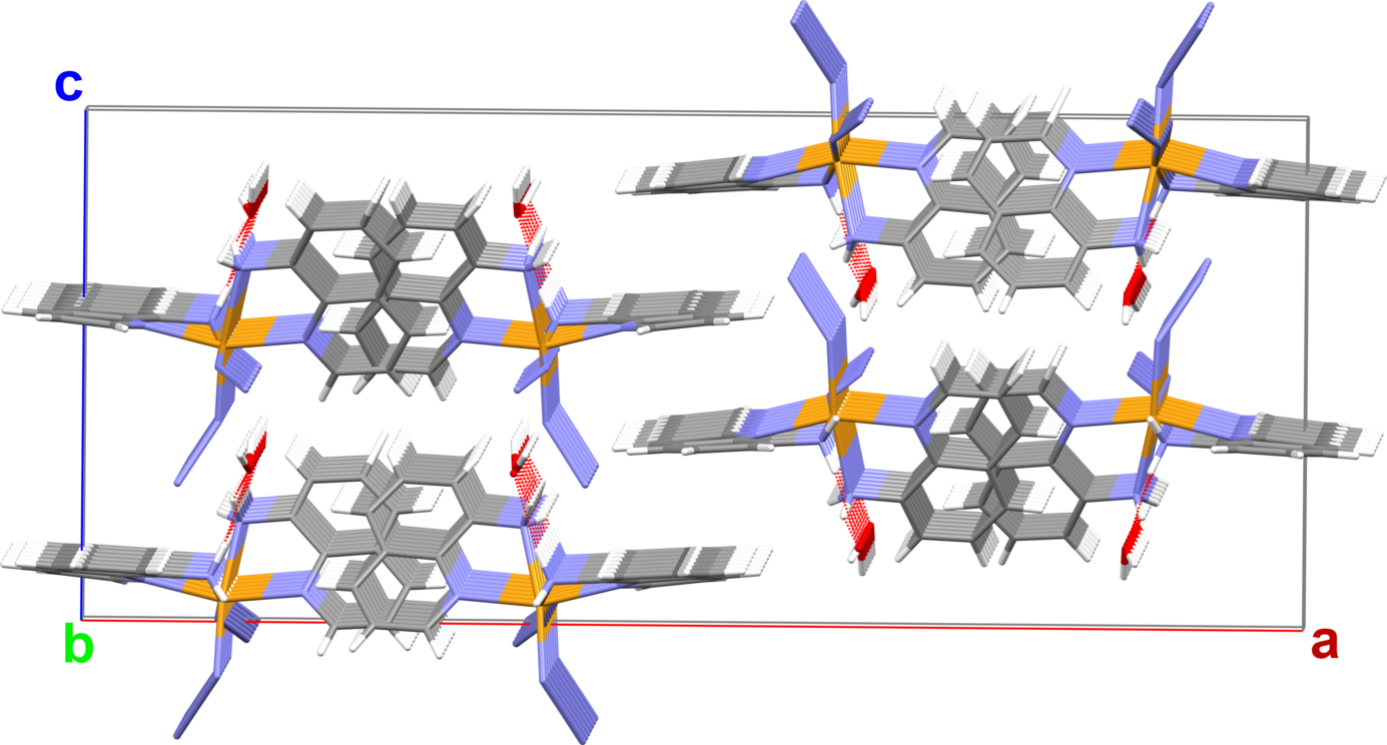
Stick model of the crystal packing down the crystallographic *b* axis. Color code: N = blue, H = white, C = gray, O = red, Fe = orange. O⋯H_2_—N hydrogen bonds between the two amine groups of each mol­ecule are shown with red dashed lines. The O—H⋯N_Azide_ hydrogen bonds linking the mol­ecules into columns parallel to the *b* axis are omitted for clarity.

In the crystal, the complex mol­ecules are arranged in columns parallel to the *b*-axis *via* N_*azide*_⋯H—O hydrogen bonds while the O⋯H_2_—N hydrogen bonds act as bridges between the two amine groups of one and the same Fe(N_3_)_2_(AQ)_2_-mol­ecule (Fig. 6 ▸).

## Synthesis and crystallization

The title compound was prepared solvothermally from a mixture of iron(II) bis­(tetra­fluoro­borate) hexa­hydrate (34 mg, 0.1 mmol), 8-amino­quinoline (29 mg, 0.2 mmol) and sodium azide (13 mg, 0.2 mmol) in a mixture of water/ethanol (4:1 *v*/*v*, 25 ml). This mixture was sealed in a Teflon-lined autoclave and held at 400 K for 2 d, and then cooled to ambient temperature at a rate of 10 K h^−1^ to give the product in form of red plates (yield 36%).

## Refinement

Crystal data, data collection and structure refinement details are summarized in Table 3 ▸.

## Supplementary Material

Crystal structure: contains datablock(s) I. DOI: 10.1107/S2414314625002354/zl4079sup1.cif

Structure factors: contains datablock(s) I. DOI: 10.1107/S2414314625002354/zl4079Isup2.hkl

CCDC reference: 2431539

Additional supporting information:  crystallographic information; 3D view; checkCIF report

## Figures and Tables

**Table 1:** Selected geometric parameters (Å, °)

Fe1—N1	2.142 (2)	Fe1—N4	2.112 (2)
N1—N2	1.159 (3)	N4—N5	1.202 (2)
N2—N3	1.166 (3)	N5—N6	1.156 (3)
			
N1—N2—N3	177.3 (3)	N4—N5—N6	179.2 (2)
N2—N1—Fe1	157.4 (2)	N5—N4—Fe1	122.3 (2)

**Table 2:** Hydrogen-bond geometry (Å, °)

*D*—H⋯*A*	*D*—H	H⋯*A*	*D*⋯*A*	*D*—H⋯*A*
O1—H1⋯N4^i^	0.96	1.88	2.838 (3)	175
O1—H2⋯N3^ii^	0.96	1.85	2.807 (3)	174
N2*A*—H11⋯N6^ii^	0.89	2.17	3.052 (3)	170
N2*A*—H12⋯O1	0.89	2.28	3.086 (3)	151
N2*B*—H21⋯N3^iii^	0.89	2.35	3.206 (3)	160
N2*B*—H22⋯O1	0.89	2.17	3.037 (2)	164

Symmetry codes: (i) 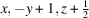; (ii) 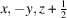; (iii) 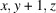.

**Table 3:** Experimental details

Crystal data	
Chemical formula	[Fe(N_3_)_2_(C_9_H_8_N_2_)_2_]·H_2_O
*M*_r_	446.27
Crystal system, space group	Orthorhombic, *Pbcn*
Temperature (K)	293
*a*, *b*, *c* (Å)	32.5164 (15), 8.8531 (5), 13.5952 (6)
*V* (Å^3^)	3913.7 (3)
*Z*	8
Radiation type	Mo *K*α
μ (mm^−1^)	0.81
Crystal size (mm)	0.27 × 0.11 × 0.05

Data collection	
Diffractometer	Bruker APEXII CCD
Absorption correction	Multi-scan (*SADABS*; Krause *et al.*, 2015 ▸)
*T*_min_, *T*_max_	0.789, 0.913
No. of measured, independent and observed [*I* > 2σ(*I*)] reflections	20186, 5690, 3470
*R*_int_	0.050
(sin θ/λ)_max_ (Å^−1^)	0.703

Refinement	
*R*[*F*^2^ > 2σ(*F*^2^)], *wR*(*F*^2^), *S*	0.047, 0.100, 0.93
No. of reflections	5690
No. of parameters	273
H-atom treatment	H-atom parameters constrained
Δρ_max_, Δρ_min_ (e Å^−3^)	0.50, −0.46

Computer programs: *APEX2* and *SAINT-Plus* (Bruker, 2019 ▸), *SHELXT* (Sheldrick, 2015*a* ▸), *SHELXL* (Sheldrick, 2015*b* ▸), *DIAMOND* (Brandenburg, 2006 ▸), *Mercury* (Macrae *et al.*, 2020 ▸) and *publCIF* (Westrip, 2010 ▸).

## full crystallographic data

### Bis(azido-κN)bis(quinolin-8-amine-κ2N,N')iron(II) monohydrate Crystal data

**Table:**

[Fe(N_3_)_2_(C_9_H_8_N_2_)_2_]·H_2_O	*D*_x_ = 1.515 Mg m^−^^3^
*M**_r_* = 446.27	Mo *K*α radiation, λ = 0.71073 Å
Orthorhombic, *P**b**c**n*	Cell parameters from 5258 reflections
*a* = 32.5164 (15) Å	θ = 2.4–28.5°
*b* = 8.8531 (5) Å	µ = 0.81 mm^−^^1^
*c* = 13.5952 (6) Å	*T* = 293 K
*V* = 3913.7 (3) Å^3^	Plate, red
*Z* = 8	0.27 × 0.11 × 0.05 mm
*F*(000) = 1840	

### Bis(azido-κN)bis(quinolin-8-amine-κ2N,N')iron(II) monohydrate Data collection

**Table:**

Bruker APEXII CCD diffractometer	5690 independent reflections
Radiation source: sealed X-ray tube	3470 reflections with *I* > 2σ(*I*)
Graphite monochromator	*R*_int_ = 0.050
φ and ω scans	θ_max_ = 30.0°, θ_min_ = 2.8°
Absorption correction: multi-scan (SADABS; Krause *et al.*, 2015)	*h* = −45→45
*T*_min_ = 0.789, *T*_max_ = 0.913	*k* = −12→8
20186 measured reflections	*l* = −19→13

### Bis(azido-κN)bis(quinolin-8-amine-κ2N,N')iron(II) monohydrate Refinement

**Table:**

Refinement on *F*^2^	Primary atom site location: structure-invariant direct methods
Least-squares matrix: full	Hydrogen site location: mixed
*R*[*F*^2^ > 2σ(*F*^2^)] = 0.047	H-atom parameters constrained
*wR*(*F*^2^) = 0.100	*w* = 1/[σ^2^(*F*_o_^2^) + (0.0476*P*)^2^] where *P* = (*F*_o_^2^ + 2*F*_c_^2^)/3
*S* = 0.93	(Δ/σ)_max_ = 0.001
5690 reflections	Δρ_max_ = 0.50 e Å^−^^3^
273 parameters	Δρ_min_ = −0.46 e Å^−^^3^
0 restraints	

### Bis(azido-κN)bis(quinolin-8-amine-κ2N,N')iron(II) monohydrate Special details

**Table:**

Geometry. All esds (except the esd in the dihedral angle between two l.s. planes) are estimated using the full covariance matrix. The cell esds are taken into account individually in the estimation of esds in distances, angles and torsion angles; correlations between esds in cell parameters are only used when they are defined by crystal symmetry. An approximate (isotropic) treatment of cell esds is used for estimating esds involving l.s. planes.
Refinement. The positions of all H atoms were clearly identified in difference Fourier syntheses. Those of the organic ligands were refined with calculated positions (–CH– = 0.93 Å, –NH_2_– = 0.89 Å) and isotropic displacement parameters depending on the equivalent isotropic temperature factor of the parent atoms. The position of the H atom of the water molecule were refined with fixed O—H distances of 0.96 Å and a bond angle of 104.95° before they were fixed and allowed to ride on the parent O atom with isotropic displacement parameters.

### Bis(azido-κN)bis(quinolin-8-amine-κ2N,N')iron(II) monohydrate Fractional atomic coordinates and isotropic or equivalent isotropic displacement parameters (Å^2^)

**Table:**

	*x*	*y*	*z*	*U*_iso_*/*U*_eq_	
Fe1	0.38035 (2)	0.21817 (4)	0.56455 (2)	0.02331 (9)	
O1	0.35856 (5)	0.4514 (2)	0.82039 (12)	0.0403 (4)	
H1	0.3663	0.5464	0.8490	0.046 (8)*	
H2	0.3573	0.3822	0.8747	0.080 (11)*	
N1	0.38357 (7)	−0.0156 (3)	0.52450 (17)	0.0498 (6)	
N2	0.37150 (5)	−0.1342 (2)	0.50298 (13)	0.0265 (4)	
N3	0.36072 (7)	−0.2560 (2)	0.48292 (19)	0.0475 (6)	
N4	0.38371 (6)	0.2776 (2)	0.41435 (13)	0.0298 (4)	
N5	0.40034 (5)	0.1988 (2)	0.35437 (13)	0.0259 (4)	
N6	0.41626 (6)	0.1243 (2)	0.29588 (15)	0.0382 (5)	
N1A	0.31339 (5)	0.2393 (2)	0.57831 (13)	0.0268 (4)	
C1A	0.28792 (7)	0.3007 (3)	0.51410 (17)	0.0325 (5)	
H1A	0.2989	0.3567	0.4626	0.039*	
C2A	0.24486 (7)	0.2857 (3)	0.51992 (18)	0.0383 (6)	
H2A	0.2280	0.3338	0.4744	0.046*	
C3A	0.22820 (7)	0.2004 (3)	0.59249 (18)	0.0363 (6)	
H3A	0.1998	0.1876	0.5960	0.044*	
C4A	0.25394 (7)	0.1311 (3)	0.66260 (16)	0.0301 (5)	
C5A	0.23947 (7)	0.0367 (3)	0.73857 (18)	0.0374 (6)	
H5A	0.2115	0.0178	0.7448	0.045*	
C6A	0.26660 (8)	−0.0267 (3)	0.80300 (19)	0.0401 (6)	
H6A	0.2570	−0.0923	0.8512	0.048*	
C7A	0.30896 (7)	0.0055 (3)	0.79768 (17)	0.0333 (5)	
H7A	0.3268	−0.0367	0.8434	0.040*	
C8A	0.32400 (6)	0.0983 (2)	0.72585 (15)	0.0258 (5)	
C9A	0.29669 (6)	0.1584 (2)	0.65421 (15)	0.0243 (5)	
N2A	0.36646 (5)	0.1420 (2)	0.71743 (13)	0.0271 (4)	
H11	0.3825	0.0640	0.7328	0.033*	
H12	0.3718	0.2166	0.7595	0.033*	
N1B	0.44530 (5)	0.2273 (2)	0.60288 (14)	0.0283 (4)	
C1B	0.47090 (7)	0.1127 (3)	0.60427 (19)	0.0395 (6)	
H1B	0.4602	0.0158	0.5973	0.047*	
C2B	0.51386 (7)	0.1293 (3)	0.61585 (19)	0.0441 (7)	
H2B	0.5308	0.0446	0.6181	0.053*	
C3B	0.53032 (7)	0.2701 (3)	0.62365 (18)	0.0380 (6)	
H3B	0.5586	0.2825	0.6297	0.046*	
C4B	0.50415 (7)	0.3966 (3)	0.62245 (15)	0.0298 (5)	
C5B	0.51836 (7)	0.5466 (3)	0.62898 (17)	0.0377 (6)	
H5B	0.5464	0.5656	0.6338	0.045*	
C6B	0.49145 (8)	0.6632 (3)	0.62832 (17)	0.0392 (6)	
H6B	0.5013	0.7617	0.6319	0.047*	
C7B	0.44891 (7)	0.6373 (3)	0.62234 (16)	0.0302 (5)	
H7B	0.4308	0.7187	0.6230	0.036*	
C8B	0.43379 (6)	0.4934 (3)	0.61555 (14)	0.0238 (5)	
C9B	0.46122 (6)	0.3697 (3)	0.61423 (14)	0.0236 (5)	
N2B	0.39027 (5)	0.4594 (2)	0.61037 (13)	0.0251 (4)	
H21	0.3782	0.5207	0.5672	0.030*	
H22	0.3788	0.4749	0.6689	0.030*	

### Bis(azido-κN)bis(quinolin-8-amine-κ2N,N')iron(II) monohydrate Atomic displacement parameters (Å^2^)

**Table:**

	*U*^11^	*U*^22^	*U*^33^	*U*^12^	*U*^13^	*U*^23^
Fe1	0.02297 (14)	0.02148 (16)	0.02548 (15)	−0.00273 (14)	−0.00115 (13)	−0.00175 (15)
O1	0.0536 (11)	0.0335 (10)	0.0338 (10)	−0.0087 (8)	−0.0002 (8)	−0.0067 (9)
N1	0.0737 (16)	0.0235 (12)	0.0521 (14)	−0.0043 (12)	−0.0006 (12)	−0.0056 (11)
N2	0.0255 (9)	0.0261 (12)	0.0279 (10)	0.0029 (8)	0.0000 (7)	0.0004 (9)
N3	0.0475 (13)	0.0320 (14)	0.0631 (16)	−0.0089 (11)	−0.0041 (12)	−0.0109 (12)
N4	0.0368 (10)	0.0269 (10)	0.0257 (10)	0.0016 (9)	0.0013 (8)	−0.0001 (9)
N5	0.0241 (9)	0.0263 (11)	0.0273 (10)	−0.0077 (8)	−0.0012 (8)	0.0022 (9)
N6	0.0432 (12)	0.0336 (13)	0.0377 (12)	−0.0038 (10)	0.0089 (9)	−0.0066 (10)
N1A	0.0256 (9)	0.0272 (11)	0.0276 (10)	−0.0035 (8)	−0.0023 (7)	0.0017 (8)
C1A	0.0333 (12)	0.0334 (15)	0.0308 (12)	0.0009 (11)	−0.0029 (10)	0.0028 (11)
C2A	0.0309 (12)	0.0454 (16)	0.0386 (13)	0.0035 (12)	−0.0093 (10)	0.0010 (13)
C3A	0.0225 (11)	0.0443 (17)	0.0420 (14)	−0.0027 (11)	−0.0023 (9)	−0.0069 (13)
C4A	0.0279 (11)	0.0295 (14)	0.0328 (12)	−0.0033 (10)	0.0015 (10)	−0.0077 (11)
C5A	0.0322 (13)	0.0398 (17)	0.0401 (14)	−0.0115 (12)	0.0090 (11)	−0.0048 (12)
C6A	0.0456 (14)	0.0380 (17)	0.0369 (14)	−0.0093 (12)	0.0102 (12)	0.0035 (13)
C7A	0.0385 (13)	0.0283 (13)	0.0332 (13)	0.0006 (11)	0.0025 (10)	0.0028 (12)
C8A	0.0291 (11)	0.0220 (12)	0.0263 (11)	−0.0025 (9)	0.0018 (9)	−0.0045 (10)
C9A	0.0266 (11)	0.0195 (11)	0.0269 (11)	−0.0031 (9)	−0.0005 (9)	−0.0031 (10)
N2A	0.0273 (9)	0.0244 (11)	0.0295 (10)	−0.0010 (8)	−0.0048 (7)	−0.0021 (9)
N1B	0.0283 (9)	0.0254 (10)	0.0312 (10)	0.0048 (9)	−0.0004 (8)	−0.0008 (9)
C1B	0.0365 (14)	0.0353 (16)	0.0467 (15)	0.0071 (12)	0.0001 (11)	0.0025 (13)
C2B	0.0319 (14)	0.0491 (19)	0.0513 (16)	0.0178 (13)	−0.0006 (12)	0.0036 (15)
C3B	0.0214 (11)	0.0588 (18)	0.0337 (13)	0.0030 (12)	−0.0001 (9)	0.0034 (13)
C4B	0.0250 (11)	0.0462 (16)	0.0183 (11)	−0.0046 (11)	−0.0018 (8)	0.0017 (11)
C5B	0.0281 (12)	0.0561 (18)	0.0288 (13)	−0.0189 (12)	−0.0016 (10)	0.0007 (12)
C6B	0.0449 (15)	0.0380 (15)	0.0349 (14)	−0.0177 (13)	−0.0025 (11)	−0.0014 (12)
C7B	0.0382 (13)	0.0256 (13)	0.0268 (12)	−0.0056 (11)	−0.0002 (9)	−0.0026 (11)
C8B	0.0258 (11)	0.0287 (12)	0.0170 (10)	−0.0054 (9)	0.0011 (8)	−0.0012 (9)
C9B	0.0236 (10)	0.0299 (13)	0.0174 (10)	−0.0044 (10)	−0.0009 (8)	−0.0002 (10)
N2B	0.0230 (9)	0.0261 (11)	0.0260 (10)	−0.0005 (8)	0.0006 (7)	0.0005 (8)

### Bis(azido-κN)bis(quinolin-8-amine-κ2N,N')iron(II) monohydrate Geometric parameters (Å, º)

**Table:**

Fe1—N1	2.142 (2)	C7A—C8A	1.367 (3)
Fe1—N1B	2.177 (2)	C7A—H7A	0.9300
Fe1—N1A	2.193 (2)	C8A—C9A	1.421 (3)
Fe1—N2A	2.231 (2)	C8A—N2A	1.438 (3)
Fe1—N2B	2.248 (2)	N2A—H11	0.8900
O1—H1	0.9600	N2A—H12	0.8900
O1—H2	0.9600	N1B—C1B	1.313 (3)
N1—N2	1.159 (3)	N1B—C9B	1.372 (3)
N2—N3	1.166 (3)	C1B—C2B	1.413 (3)
Fe1—N4	2.112 (2)	C1B—H1B	0.9300
N4—N5	1.202 (2)	C2B—C3B	1.361 (4)
N5—N6	1.156 (3)	C2B—H2B	0.9300
N1A—C1A	1.321 (3)	C3B—C4B	1.407 (3)
N1A—C9A	1.368 (3)	C3B—H3B	0.9300
C1A—C2A	1.409 (3)	C4B—C5B	1.409 (3)
C1A—H1A	0.9300	C4B—C9B	1.421 (3)
C2A—C3A	1.356 (3)	C5B—C6B	1.353 (4)
C2A—H2A	0.9300	C5B—H5B	0.9300
C3A—C4A	1.409 (3)	C6B—C7B	1.405 (3)
C3A—H3A	0.9300	C6B—H6B	0.9300
C4A—C5A	1.409 (3)	C7B—C8B	1.369 (3)
C4A—C9A	1.415 (3)	C7B—H7B	0.9300
C5A—C6A	1.364 (3)	C8B—C9B	1.412 (3)
C5A—H5A	0.9300	C8B—N2B	1.449 (2)
C6A—C7A	1.409 (3)	N2B—H21	0.8900
C6A—H6A	0.9300	N2B—H22	0.8900
			
N4—Fe1—N1	89.57 (8)	C9A—C8A—N2A	116.40 (19)
N4—Fe1—N1B	99.91 (7)	N1A—C9A—C4A	122.67 (19)
N1—Fe1—N1B	92.82 (8)	N1A—C9A—C8A	117.69 (18)
N4—Fe1—N1A	96.47 (7)	C4A—C9A—C8A	119.6 (2)
N1—Fe1—N1A	98.77 (8)	C8A—N2A—Fe1	110.46 (12)
N1B—Fe1—N1A	159.98 (7)	C8A—N2A—H11	109.6
N4—Fe1—N2A	170.62 (7)	Fe1—N2A—H11	109.6
N1—Fe1—N2A	87.41 (8)	C8A—N2A—H12	109.6
N1B—Fe1—N2A	89.12 (7)	Fe1—N2A—H12	109.6
N1A—Fe1—N2A	75.25 (6)	H11—N2A—H12	108.1
N4—Fe1—N2B	91.37 (7)	C1B—N1B—C9B	118.0 (2)
N1—Fe1—N2B	168.84 (8)	C1B—N1B—Fe1	126.16 (17)
N1B—Fe1—N2B	76.06 (7)	C9B—N1B—Fe1	115.29 (14)
N1A—Fe1—N2B	92.18 (6)	N1B—C1B—C2B	123.2 (3)
N2A—Fe1—N2B	93.32 (7)	N1B—C1B—H1B	118.4
H1—O1—H2	105.0	C2B—C1B—H1B	118.4
N1—N2—N3	177.3 (3)	C3B—C2B—C1B	119.5 (2)
N2—N1—Fe1	157.4 (2)	C3B—C2B—H2B	120.2
N4—N5—N6	179.2 (2)	C1B—C2B—H2B	120.2
N5—N4—Fe1	122.3 (2)	C2B—C3B—C4B	119.4 (2)
C1A—N1A—C9A	117.74 (19)	C2B—C3B—H3B	120.3
C1A—N1A—Fe1	126.96 (16)	C4B—C3B—H3B	120.3
C9A—N1A—Fe1	114.43 (13)	C3B—C4B—C5B	123.5 (2)
N1A—C1A—C2A	123.2 (2)	C3B—C4B—C9B	117.5 (2)
N1A—C1A—H1A	118.4	C5B—C4B—C9B	119.0 (2)
C2A—C1A—H1A	118.4	C6B—C5B—C4B	120.4 (2)
C3A—C2A—C1A	119.4 (2)	C6B—C5B—H5B	119.8
C3A—C2A—H2A	120.3	C4B—C5B—H5B	119.8
C1A—C2A—H2A	120.3	C5B—C6B—C7B	120.9 (2)
C2A—C3A—C4A	119.8 (2)	C5B—C6B—H6B	119.6
C2A—C3A—H3A	120.1	C7B—C6B—H6B	119.6
C4A—C3A—H3A	120.1	C8B—C7B—C6B	120.6 (2)
C5A—C4A—C3A	123.7 (2)	C8B—C7B—H7B	119.7
C5A—C4A—C9A	119.2 (2)	C6B—C7B—H7B	119.7
C3A—C4A—C9A	117.0 (2)	C7B—C8B—C9B	119.7 (2)
C6A—C5A—C4A	119.9 (2)	C7B—C8B—N2B	123.2 (2)
C6A—C5A—H5A	120.0	C9B—C8B—N2B	117.08 (19)
C4A—C5A—H5A	120.0	N1B—C9B—C8B	118.40 (18)
C5A—C6A—C7A	121.1 (2)	N1B—C9B—C4B	122.3 (2)
C5A—C6A—H6A	119.5	C8B—C9B—C4B	119.3 (2)
C7A—C6A—H6A	119.5	C8B—N2B—Fe1	110.56 (13)
C8A—C7A—C6A	120.6 (2)	C8B—N2B—H21	109.5
C8A—C7A—H7A	119.7	Fe1—N2B—H21	109.5
C6A—C7A—H7A	119.7	C8B—N2B—H22	109.5
C7A—C8A—C9A	119.4 (2)	Fe1—N2B—H22	109.5
C7A—C8A—N2A	124.2 (2)	H21—N2B—H22	108.1
			
C9A—N1A—C1A—C2A	−0.6 (3)	C9B—N1B—C1B—C2B	−0.6 (4)
Fe1—N1A—C1A—C2A	168.09 (18)	Fe1—N1B—C1B—C2B	170.49 (18)
N1A—C1A—C2A—C3A	−2.2 (4)	N1B—C1B—C2B—C3B	−1.7 (4)
C1A—C2A—C3A—C4A	1.6 (4)	C1B—C2B—C3B—C4B	1.5 (4)
C2A—C3A—C4A—C5A	−177.9 (2)	C2B—C3B—C4B—C5B	−179.3 (2)
C2A—C3A—C4A—C9A	1.5 (3)	C2B—C3B—C4B—C9B	0.8 (3)
C3A—C4A—C5A—C6A	179.5 (2)	C3B—C4B—C5B—C6B	−179.4 (2)
C9A—C4A—C5A—C6A	0.1 (3)	C9B—C4B—C5B—C6B	0.6 (3)
C4A—C5A—C6A—C7A	2.8 (4)	C4B—C5B—C6B—C7B	0.8 (4)
C5A—C6A—C7A—C8A	−1.8 (4)	C5B—C6B—C7B—C8B	−1.0 (3)
C6A—C7A—C8A—C9A	−2.1 (3)	C6B—C7B—C8B—C9B	−0.2 (3)
C6A—C7A—C8A—N2A	177.4 (2)	C6B—C7B—C8B—N2B	179.2 (2)
C1A—N1A—C9A—C4A	4.0 (3)	C1B—N1B—C9B—C8B	−178.3 (2)
Fe1—N1A—C9A—C4A	−166.08 (17)	Fe1—N1B—C9B—C8B	9.6 (2)
C1A—N1A—C9A—C8A	−177.1 (2)	C1B—N1B—C9B—C4B	3.1 (3)
Fe1—N1A—C9A—C8A	12.9 (2)	Fe1—N1B—C9B—C4B	−168.97 (15)
C5A—C4A—C9A—N1A	175.0 (2)	C7B—C8B—C9B—N1B	−177.09 (19)
C3A—C4A—C9A—N1A	−4.5 (3)	N2B—C8B—C9B—N1B	3.4 (3)
C5A—C4A—C9A—C8A	−3.9 (3)	C7B—C8B—C9B—C4B	1.6 (3)
C3A—C4A—C9A—C8A	176.6 (2)	N2B—C8B—C9B—C4B	−177.91 (17)
C7A—C8A—C9A—N1A	−174.1 (2)	C3B—C4B—C9B—N1B	−3.2 (3)
N2A—C8A—C9A—N1A	6.4 (3)	C5B—C4B—C9B—N1B	176.8 (2)
C7A—C8A—C9A—C4A	4.9 (3)	C3B—C4B—C9B—C8B	178.22 (19)
N2A—C8A—C9A—C4A	−174.59 (19)	C5B—C4B—C9B—C8B	−1.7 (3)
C7A—C8A—N2A—Fe1	158.94 (18)	C7B—C8B—N2B—Fe1	166.65 (17)
C9A—C8A—N2A—Fe1	−21.6 (2)	C9B—C8B—N2B—Fe1	−13.9 (2)

### Bis(azido-κN)bis(quinolin-8-amine-κ2N,N')iron(II) monohydrate Hydrogen-bond geometry (Å, º)

**Table:**

*D*—H···*A*	*D*—H	H···*A*	*D*···*A*	*D*—H···*A*
O1—H1···N4^i^	0.96	1.88	2.838 (3)	175
O1—H2···N3^ii^	0.96	1.85	2.807 (3)	174
N2*A*—H11···N6^ii^	0.89	2.17	3.052 (3)	170
N2*A*—H12···O1	0.89	2.28	3.086 (3)	151
N2*B*—H21···N3^iii^	0.89	2.35	3.206 (3)	160
N2*B*—H22···O1	0.89	2.17	3.037 (2)	164

Symmetry codes: (i) *x*, −*y*+1, *z*+1/2; (ii) *x*, −*y*, *z*+1/2; (iii) *x*, *y*+1, *z*.
